# The Added Burden of Transfer Status in Patients Undergoing Surgery After Sustaining a Periprosthetic Fracture of the Hip or Knee

**DOI:** 10.7759/cureus.16805

**Published:** 2021-08-01

**Authors:** Edwin Chaharbakhshi, Daniel Schmitt, Nicholas M Brown

**Affiliations:** 1 Orthopaedic Surgery, West Virginia University, Morgantown, USA; 2 Orthopaedic Surgery, Loyola University Medical Center, Maywood, USA; 3 Orthopaedic Surgery & Rehabilitation, Loyola University Medical Center, Maywood, USA

**Keywords:** arthroplasty, periprosthetic fracture, transfer, readmission, complication

## Abstract

Background

A significant proportion of patients who sustain periprosthetic fractures are transferred to another institution for definitive care. There is limited understanding of the impact of transfer on outcomes.

Methods

The American College of Surgeons National Surgical Quality Improvement Program (NSQIP) was utilized to identify patients who sustained periprosthetic fractures of the hip or knee requiring surgical intervention. Inclusion criteria were total joint prosthesis of the hip or knee with documented periprosthetic fracture of a single hip or knee along with surgery during hospitalization. Transfer status and discharge location were recorded. A total of 1,194 non-transferred patients were compared to 620 transferred patients. Cohorts were compared utilizing standard parametric and non-parametric tests depending on the characteristics of the data.

Results

Transferred patients had a higher mean age (75.6 years vs. 72.9 years, p < 0.0001), longer mean length of stay (7.9 days vs. 6.9 days, p = 0.0023), and greater American Society of Anesthesiologists (ASA) grade. Transferred patients were less likely to be discharged home (p = 0.0001) and more likely to be discharged to hospice (p = 0.049) or rehabilitation facilities (p = 0.0001). No significant differences were detected regarding readmissions or complications. In transferred patients, having lower preoperative albumin was a risk factor for readmission within 30 days.

Conclusion

Transfer centers accepting and treating periprosthetic fractures should be aware that these patients often have a longer length of stay and are less likely to be discharged home. However, the data suggests these patients are well cared for, given the similar complication rates.

## Introduction

Interfacility patient transfers are a significant logistical undertaking dependent on factors such as handoffs, procedures, risk stratification, and unpredictability [1,2]. There is a breadth of literature evaluating the potentially negative effects of transfer status on patient outcomes across disciplines [3-7]. A recent study by Mueller et al. reported that between 32% and 89% of transferred patients did not receive specialty care despite being transferred from an acute care hospital, suggesting that a significant proportion of transfers may not have been appropriate [3]. Overall, inappropriate patient transfers can accumulate significant burdens in cost and resource allocation to healthcare systems worldwide.

There has been a growing body of research over the past decade exploring orthopedic trauma transfers. O’Connell et al. demonstrated that up to 20% of orthopedic trauma transfers at a level I trauma center were inappropriate [8]. This observation was even larger in Thakur’s study, which reported that up to 52% of orthopedic trauma transfers were inappropriate [9]. In terms of risk, a recent study by Boyd et al. demonstrated that patients with lower extremity fractures who were subsequently transferred had a 9.5% risk of developing venous thromboembolism, compared to 0.7% in non-transferred patients [10]. Importantly, some of the findings in recent literature may be related to the observation that only a limited number of patients are examined by an orthopedic surgeon prior to transfer [11].

Given that many periprosthetic fracture patients undergo transfer, it is important to determine whether these patients differed from non-transferred patients in terms of demographics, comorbidities, and outcomes. A more definitive answer to this question may allow physicians and health systems to better anticipate appropriate levels of care and understand the impact of treating these complex patients. We hypothesized that patients who required transfers to another facility would have more comorbidities, perioperative complications, and would be less likely to be discharged home from the hospital.

## Materials and methods

Data source

The authors of the present study requested and were granted access to the American College of Surgeons National Surgical Quality Improvement Program (NSQIP) database. This database is a nationally validated tool that has been previously used to assist in improving surgical care in general. The NSQIP database contained various data for surgical cases that occurred between 2015 and 2018. Of note, the data analysis is available for sharing in the form of a Microsoft Excel (Microsoft Corporation, Redmond, Washington, United States) and/or MiniTab spreadsheet.

Study design

The NSQIP database (cases between 2015 and 2018) was used as the sole source of data. The present study was designed as a retrospective cohort study that compared patients who sustained a single periprosthetic fracture of the hip or knee, were hospitalized, and were either transferred to a new facility or not transferred for surgical intervention of that periprosthetic fracture. Inclusion criteria were patients who had a pre-existing total joint prosthesis of the hip or knee, had a documented periprosthetic fracture of the hip or knee, underwent surgical intervention during the same hospitalization, had a transfer status documented (non-transfers vs. transfers), and had a discharge location documented. Exclusion criteria were patients who had more than one affected prosthetic joint or required any other surgical interventions during the total hospitalization period.

Data utilized

Patient demographic variables, comorbidities, preoperative laboratory values, perioperative complications (stroke, myocardial infarction, pneumonia, surgical site infection, pulmonary embolism, urinary tract infection), readmissions, and discharge destination were used. Although initially requested, insurance information and specific surgical dates were not available to NSQIP users to help ensure patient confidentiality. After applying the aforementioned inclusion and exclusion criteria, the desired data was isolated into a separate file for statistical analysis.

Statistical analysis

All analyses were performed using Microsoft Excel and MiniTab. Descriptive statistics including mean, standard deviation, ranges, and proportions were calculated and reported when appropriate. The Shapiro-Wilk test was performed to assess continuous data for normal distributions. The F-test was performed to demonstrate which continuous data demonstrated equal variances. For data following a normal distribution, the two-tailed independent samples t-test was performed to compare mean differences between groups. If not normally distributed, the Mann-Whitney U test was utilized. The Chi-squared and Fisher's exact tests were performed to assess for differences in proportions between groups when using categorical data. A multiple linear regression was performed with forced entry (the enter method) to identify the odds of 30-day readmission based on the preoperative laboratory values housed in NSQIP. Odds ratios and confidence intervals that met the threshold for statistical significance (p < 0.05) were reported.

## Results

Patient demographics

After isolating patients who sustained periprosthetic fractures of the hip or knee, a group of 1194 patients who did not require transfer were compared to a group of 620 patients who required transfer prior to surgical intervention. Comparisons of demographics are detailed in Table 1. Patients who required transfers were, on average, older (75.6 years vs. 72.9 years, p < 0.0001), more likely to be male (35.0% vs. 29.3%, p = 0.0132), more likely to have a periprosthetic hip as compared to knee fracture (78.1% vs. 69.6%, p = 0.0001), and had a longer time from the initial hospital’s admission to eventual discharge (7.9 days vs. 6.9 days, p = 0.0023). No statistically significant differences were observed between the two groups regarding ethnicity or mean body mass index (BMI).

**Table 1:** Patient demographics

Patient demographics	Non-transfers	Transfers	P-value
Patients (n)	1194	620	
Age in years (mean, SD)	72.9 (10.9)	75.6 (10.7)	< 0.0001
Gender (n%)			0.0132
Male	350 (29.3%)	217 (35%)	
Female	844 (70.7%)	403 (65%)	
Ethnicity (n%)			0.75
American Indian	6 (.5%)	6 (.97%)	
Asian	18 (1.5%)	7 (1.1%)	
Black or African American	55 (4.6%)	28 (4.5%)	
White	910 (76.2%)	468 (75.5%)	
Unreported	205 (17.2%)	111 (17.9%)	
Body mass index (mean, SD)	29.1 (7.2)	28.5 (7.9)	0.104
Periprosthetic knee fractures (n%)	363 (30.4%)	136 (21.9%)	0.0001
Periprosthetic hip fractures (n%)	831 (69.6%)	484 (78.1%)	0.0001
Days from admission to discharge (mean, SD)	6.9 (6.6)	7.9 (6.9)	0.0023

Preoperative comorbidities

Comparisons of preoperative comorbidities are detailed in Table 2. Patients who were transferred were more likely to have an ongoing malignancy (3.6% vs. 1.1%, p = 0.03). In regards to ASA classification, transferred patients were more likely to have an ASA grade of 4 (21.5% vs. 11.8%, p = 0.0001), while not statistically significant transferred patients were more likely to have heart failure (11.4% vs. 8.1%, p = 0.06).

Patients who did not require transfer were more likely to have an ASA grade of 1 (1.0% vs. 0%, p = 0.011) or 2 (21.8% vs. 13.4%, p = 0.0001). There were no other statistical differences or trends observed regarding smoking status or other major preoperative comorbidities.

**Table 2:** Preoperative comorbidities

Preoperative comorbidities	Non-transfers	Transfers	P-value
Smoking history (n%)	130 (10.9%)	57 (9.2%)	0.29
Comorbidities present (n)	356	220	
Diabetes (n%)	221 (62.1%)	123 (55.9%)	0.49
Heart failure (n%)	29 (8.1%)	25 (11.4%)	0.06
Renal failure (n%)	3 (0.8%)	4 (1.8%)	0.24
Malignancy (n%)	4 (1.1%)	8 (3.6%)	0.03
Chronic obstructive pulmonary disease (n%)	97 (27.2%)	60 (27.3%)	0.29
Ascites (n%)	2 (0.5%)	0	0.55
ASA classification (n%)			
1	12 (1.0%)	0	0.011
2	260 (21.8%)	83 (13.4%)	0.0001
3	778 (65.2%)	404 (65.2%)	1
4	141 (11.8%)	133 (21.5%)	0.0001

Preoperative laboratory values

Comparisons of preoperative laboratory values are detailed in Table 3. Patients who required transfers tended to have a higher mean creatinine (1.03 vs. 0.93, p = 0.005), lower mean albumin (3.36 vs. 3.57, p < 0.0001), lower mean hematocrit (32.39 vs. 34.61, p < 0.0001), and higher mean white blood cell count (8.94 vs. 8.61, p = 0.034). A multiple logistic regression model controlling for age, sex, and BMI demonstrated that having a lower preoperative albumin (i.e., < 3.5 g/dL) was a risk factor for readmission within 30 days of discharge in patients who required transfers (odds ratio = 1.29, 95% confidence interval = [1.068-1.44], p = 0.002).

**Table 3:** Preoperative laboratory values

Preoperative Laboratory Values	Non-transfers	Transfers	P-value
Creatinine (mean, SD)	0.93 (0.56)	1.03 (0.91)	0.005
Albumin (mean, SD)	3.57 (0.54)	3.36 (0.57)	< 0.0001
Hematocrit (mean, SD)	34.61 (5.49)	32.39 (5.10)	< 0.0001
White blood cell count (mean, SD)	8.61 (3.18)	8.94 (3.27)	0.034

Complications and readmissions

Perioperative complications and readmissions within 30 days of discharge are detailed in Table 4. No differences were observed regarding readmission rates between the non-transferred (8.3%) and transferred (7.9%) patients. Transferred patients had a longer mean operative time (154.1 minutes vs. 145.9 minutes, p = 0.014). The major complication rate between groups was 9.6% in the non-transferred group and 11.5% in the transferred group (p = 0.25). No significant differences were observed between groups when comparing individual complications.

**Table 4:** Perioperative complications and readmissions

Perioperative complications and readmissions	Non-transfers	Transfers	P-value
Operative time in minutes (mean, SD)	145.9 (66.2)	154.1 (66.9)	0.014
Readmitted within 30 days (n%)	99 (8.3%)	49 (7.9%)	0.86
Complications present (n%)	115 (9.6%)	71 (11.5%)	0.25
Pulmonary embolism (n%)	10 (0.8%)	6 (0.9%)	0.79
Myocardial infarction (n%)	18 (1.5%)	12 (2.6%)	0.56
Pneumonia (n%)	20 (1.7%)	16 (2.6%)	0.21
Cerebrovascular accident (n%)	3 (0.3%)	5 (0.8%)	0.13
Wound infection (n%)	22 (1.8%)	8 (1.3%)	0.44
Superficial infections (n%)	15 (1.3%)	5 (0.8%)	0.75
Deep infections (n%)	30 (2.5%)	8 (1.3%)	0.08
Urinary tract infection (n%)	42 (3.5%)	24 (3.9%)	0.69

Discharge destination

Comparisons of discharge destinations are detailed in Table 5. Patients who were transferred were less likely to be discharged home (19.4% vs. 32.2%, p = 0.0001), more likely to be transferred to hospice (0.7% vs. 0.08%, p = 0.049), and more likely to be transferred to be transferred to a short-term or long-term rehabilitation facility (77.4% vs. 65.7%, p = 0.0001).

**Table 5:** Discharge destination

Discharge Destination	Non-transfers	Transfers	P-value
Against medical advice (n%)	3 (0.3%)	0	0.55
Expired (n%)	21 (1.8%)	16 (2.6%)	0.29
Home (n%)	385 (32.2%)	120 (19.4%)	0.0001
Hospice (n%)	1 (0.08%)	4 (0.7%)	0.049
Rehabilitation facility (n%)	784 (65.7%)	480 (77.4%)	0.0001

## Discussion

The present study aimed to elucidate the effects, if any, on interfacility transfer in patients who sustained a periprosthetic fracture of the hip or knee. Briefly, the analysis demonstrated that transferred patients had a longer length of stay, tended to be older, and were less likely to be healthy based on the ASA classification. Additionally, transferred patients were less likely to be discharged directly home, as almost 80% required discharge to a rehabilitation facility. Although hypothesized as otherwise, there were no differences between groups regarding readmission rates or perioperative complications. Lastly, lower preoperative albumin was a risk factor for readmission within 30 days of discharge in patients who were transferred.

Most of the prior literature in orthopedic trauma has emphasized the effects of patient factors on surgical outcomes. However, there has been growing interest in the effects of the pre-hospital environment on surgical outcomes. Although a significant proportion of inappropriate orthopedic trauma transfers have been previously documented, the short-term effects of transfer status have been limited to a single study. Recently, a study used the NSQIP database to determine if transfer status was associated with increased morbidity and mortality in the geriatric population with non-periprosthetic hip fractures, and similar to our results, it also showed that transfers were associated with a significantly lower proportion of home discharges. In addition, our study demonstrated that transferred patients had a longer overall mean hospitalization time (7.9 days vs. 6.9 days). This may be associated with a longer time to surgery, which has been previously shown to be associated with increased mortality, readmissions, and perioperative complications. Overall, the results of the present study indicate an urgency to establish a risk stratification and transfer algorithm to optimize the outcomes in this complex patient population. 

Although the literature is limited, the ASA classification has been previously shown to influence outcomes in patients transferred between facilities. A recent study by Lalonde et al. showed that patients who took more medications trended toward having a higher chance of readmission [12]. Relatedly, the present study demonstrated that ASA classification is associated with transfer status, i.e., patients who were transferred tended to have an ASA grade 4, whereas non-transferees were more likely to have an ASA grade of 1 or 2. Overall, these findings are intuitive in that they suggest less-healthy patients may require higher levels of care that are not readily available at the hospital to which the patient first presents. Future studies may consider elucidating the relationship, if any, between ASA classification and the appropriateness of patient transfers.

The present study demonstrated that lower albumin levels in transferred patients were associated with increased 30-day readmission rates. In the orthopedics literature, hypoalbuminemia has been previously shown to be associated with a longer length of hospital stay, inferior clinical outcomes, higher mortality, and more postoperative complications as compared to patients with albumin levels within the normal range [13-16]. Most recently, a study by Kishawi et al. showed that patients with lower preoperative albumin levels who underwent total joint arthroplasty had increased risks of major postoperative complications and a higher 30-day readmission rate [17]. Ryan et al. compared preoperative albumin to ASA score and showed that both were significant predictors of death, superficial infections, pneumonia, renal insufficiency, reintubation, transfusion, readmissions, and reoperations; however, hypoalbuminemia was more robust in predicting deep wound infections in total hip arthroplasty and superficial wound infections in total knee arthroplasty [15]. In the context of our results, these trends further suggest that health and nutritional status in the forms of ASA classification and preoperative albumin may be important risk stratification tools when decision-making regarding patient transfers.

The present study has limitations to consider. First, given the nature of this database study, we were unable to elucidate the complexity of the periprosthetic fractures themselves. This is important to consider, as we were unable to determine if the transfers were performed because of the patient’s health care history and overall medical complexity, the complexity of the fracture itself, or a combination of the two. Second, the time between initial patient presentation at the outside hospital to transfer was not available in the NSQIP database. This may have been a confounding variable that was not accounted for in the data analysis. Third, the database was limited to complication rates within 30 days. Long-term outcomes or readmission rates were not collected.

## Conclusions

Transfer centers accepting and treating periprosthetic fractures should be aware that these patients often have a longer length of stay and are less likely to be discharged home. However, the data suggests these patients are well cared for, given the similar complication and readmission rates.
